# A New Analytical Large-Signal Model for Quasi-Ballistic Transport in InGaAs HEMTs Accommodating Dislocation Scattering

**DOI:** 10.3390/mi14051023

**Published:** 2023-05-10

**Authors:** Jinye Wang, Jun Liu, Jie Wang, Zhenxin Zhao

**Affiliations:** Zhejiang Key Laboratory of Large-Scale Integrated Circuit Design, Hangzhou Dianzi University, Hangzhou 310018, China; 191040065@hdu.edu.cn (J.W.); wangjie@hdu.edu.cn (J.W.); zhao_zhenxin@hdu.edu.cn (Z.Z.)

**Keywords:** quasi-ballistic transport, large-signal model, InGaAs HEMT, dislocation scattering, one-flux, transmission coefficient

## Abstract

A surface-potential-based analytical large-signal model, which is applicable to both ballistic and quasi-ballistic transport in InGaAs high electron mobility transistors, is developed. Based on the one-flux method and a new transmission coefficient, a new two-dimensional electron gas charge density is derived, while the dislocation scattering is novelly taken into account. Then, a unified expression for $E_{f}$ valid in all the regions of gate voltages is determined, which is utilized to directly calculate the surface potential. The flux is used to derive the drain current model incorporating important physical effects. Moreover, the gate-source capacitance $C_{gs}$ and gate-drain capacitance $C_{gd}$ are obtained analytically. The model is extensively validated with the numerical simulations and measured data of the InGaAs HEMT device with the gate length of 100 nm. The model is in excellent agreement with the measurements under I-V, C-V, small-signal conditions, and large-signal conditions.

## 1. Introduction

With its high frequency, high efficiency, high gain and low noise, InGaAs HEMT allows for low noise amplifier (LNA) and power amplifier (PA) performance. The most straightforward way to improve performance is to reduce the gate length. As the continuous scaling down of InGaAs high electron mobility transistors (HEMTs), the transport is no longer purely drift-diffusive. When the channel lengths of the devices become comparable to or smaller than the carrier mean-free-path (MFP), λ [1], new physical phenomena, such as the fully ballistic (carriers experience no scattering) or quasi-ballistic regime (carriers experience some scattering), emerge. The quasi-ballistic transport is strongly affected by the scattering events occurring during the transport from the source to the drain, especially those close to the so-called virtual source (VS). A detailed understanding of the influence of scattering is important as it is crucial in determining the on-state current of quasi-ballistic InGaAs HEMT devices. The mechanism of dislocation scattering is found in the III–V HEMT heterojunctions. Dislocation scattering causes the low lateral mobility of III–V HEMT devices and the non-monotonicity of mobility that increases first and then decreases with the increase of doping concentration. When the dislocation density is high, the dislocation scattering is the dominant mobility-degraded mechanism over other elastic scattering mechanisms and may severely limit the maximum drain current. In this context, it is necessary to develop a new analytical large signal model for quasi-ballistic transport in InGaAs HEMTs incorporating dislocation scattering.

The Advanced Spice Model (ASM) surface-potential compact model [2] for HEMT is the industry standard model. However, ASM is based on the conventional drift-diffusion theory without considering quasi-ballistic transport, as is the model proposed by Khandelwal et al. [3]. The outstanding advantage of flux theory is a technique suitable for systems with channel lengths in the order of or smaller than the mean free carrier path [4]. The method describes the transport property in terms of a single parameter: the transmission or backscattering coefficient. The existing ballistic/quasi-ballistic transport models include scattering matrix-based [5], virtual source-based [6,7], and backscattering coefficient-based [4,8] works. These models mainly deal with Si devices, especially double gated devices. The basic MIT virtual source-based model for quasi-ballistic transport in HEMTs [6,7] is presented using an empirical channel charge model. Its further extension version proposed by Rakheja et al. [9,10] does not take dislocation scattering into account. Therefore, this paper presents a novel surface-potential-based analytical large-signal model for quasi-ballistic InGaAs HEMTs using one-flux method and incorporating the dislocation scattering.

Unlike the surface-potential-based ASM-HEMT model, the two-dimensional electron gas charge density (2DEG) is derived using the one-flux method. The transmission coefficient is introduced to quantify and characterize the quasi-ballisticity. Meanwhile, dislocation scattering at the heterojunction interface is incorporated into the transmission coefficient model. Subsequently, using the proposed new charge model, the surface potential solution is calculated. From it, a compact drain current model, including channel length modulation, mobility degradation, velocity saturation, drain induced barrier reduction (DIBL), self-heating and access resistance, is established, which captures various physics of actual devices. The calculation terminal charges and capacitances are subsequently presented. The $E_{f}$ model is first validated by the numerical simulations. The drain and capacitance model and small- and large-signal model are also validated against experimental data of 100 nm InGaAs HEMT.

## 2. Model Development

In the following subsections, the InGaAs HEMT device is first described. Then, a new analytical large-signal model is proposed for InGaAs HEMT working in the quasi-ballistic regime. The model is based on the analytical calculation of Surface Potential (SP). Core model formulation containing 2DEG, surface potential (SP), current, and charges are presented.

### 2.1. InGaAs HEMT Devices

**Figure 1:**
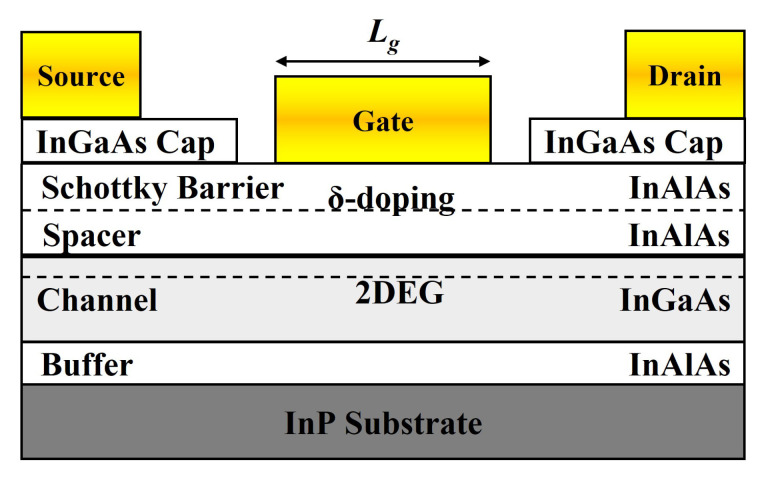
Cross-sectional view of InGaAs HEMT.

The InGaAs HEMTs fabricated in this paper are used as PA. A cross-sectional view of the InGaAs HEMT is shown in Figure 1. From bottom to top, the device structure consists of substrate, buffer, channel, δ-doping, spacer, Schottky barrier and cap layer. The InAlAs buffer layer is grown on a semi insulating InP substrate to alleviate the stress caused by lattice mismatch between the substrate and the channel. The channel layer is InGaAs, a narrow-band material, while the spacer layer is made of wide bandgap material InAlAs. The two materials with different band gap widths are in contact to form a heterojunction and a two-dimensional electron gas is formed at the interface near the channel layer. The spacer layer both increases the carrier mobility of the channel layer and reduces device noise. The spacer, δ-doping and Schottky barrier layer are the same semiconductor material. A heavily doped InGaAs is used as the cap layer to form an ohmic contact without significant additional impedance.

In InGaAs HEMT devices, the applied gate voltage regulates the built-in electric field of the heterojunction, which in turn changes the concentration of two-dimensional electron gas in the channel for the purpose of controlling current. When the gate voltage is less than the threshold voltage, the 2DEG in the channel is completely depleted and the channel is turned off. When the applied gate voltage is larger than the threshold voltage, the 2DEG in the channel increases with increasing gate voltage until saturation.

### 2.2. A New 2DEG Charge Density Model

**Figure 2:**
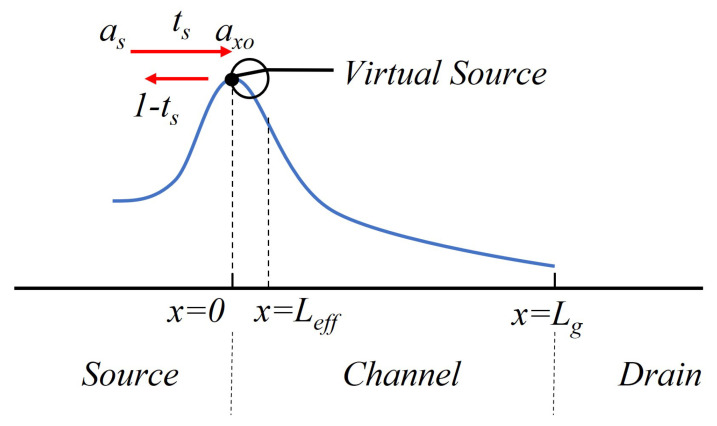
Schematic of the model framework.

When the effective gate length $L_{g}$ is less than or equal to λ, the one-flux theory is used. As shown in Figure 2, electron flux is thermionically emitted to the top of the barrier, which is the so-called virtual source (VS). A fraction ts flows out of the drain and comprises the steady-state drain current, and a fraction 1-$t_{s}$ backscatters and returns to the source. Scattering events near the VS which contribute to 1-$t_{s}$ are the most effective in controlling the on-current. The flux *a*${}_{x0}$ that constitutes the steady-state drain current at the VS is

(1) $$a_{x0}=a_{s}t_{s}$$

The injected source flux as can be calculated as [9]

(2) $$a_{s}=v_{T}N_{2-D}\sum_{i=0,1}\ln\left[1+e^{\left(E_{f}-E_{i}\right)/V_{th}}\right]$$

$E_{f}$ is the Fermi level in the source contact, $E_{0}$ and $E_{1}$ are the first and the second energy sub-bands, respectively, when *i* is taken as 0 and 1. $V_{th}$ = $k_{B}$T/q is the thermal voltage. $k_{B}$ is the Boltzmann constant. *T* denotes the absolute temperature. *q* is the electron charge. $v_{T}$ is the thermal velocity of carriers with $v_{T}$ = $\sqrt{2k_{B}T/\pi m^{*}}$. $m^{*}$ = 0.035 $m_{0}$ with $m^{*}$ is the low effective carrier mass and $m_{0}$ is the mass of electrons. $N_{2-D}$ is the effective density of states and $N_{2-D}=k_{B}Tg_{2D}$. $g_{2D}$ is the density of states and $g_{2D}$ = $2m^{*}/\pi \hbar^{2}$. *ℏ* is the approximate Planck constant.

The charge density $n_{x0}$ at the VS is inferred from the flux $a_{x0}$

(3) $$n_{x0}=t_{s}a_{x0}/v_{T}$$

Then, the 2DEG charge density ns along the channel is

(4) $$n_{s}=\int_{0}^{y_{\max}}n_{x0}dy=t_{s}N_{2-D}\sum_{i=0,1}\ln\left[1+e^{\left(E_{f}-E_{i}\right)/V_{th}}\right]$$

where $y_{max}$ is the depth of the 2DEG.

According to [9], the transmission coefficient, $t_{s}$, is functionally linked to both the MFP λ and the critical length $L_{eff}$ of the low-field region near the VS as

(5) $$t_{s}=\frac{\lambda }{L_{eff}+\lambda }=\frac{\lambda }{\kappa L_{g}}$$

For compact modeling, $L_{eff}+\lambda =\kappa L_{g}$ is obtained semi-empirically. κ is the empirical factor. λ is given as follow

(6) $$\lambda =2V_{th}\mu /v_{T}$$

where μ is the carrier mobility. According to Mathiessen’s rule, various scattering mechanisms are independent. Only dislocation scattering is considered here, while other scattering mechanisms are incorporated in the subsequent drain current model. μ considering dislocation scattering is expressed as [11]

(7) $$\mu =\mu_{dsl}=\frac{30\sqrt{2\pi }\varepsilon^{2}d_{l}^{2}{\left(k_{B}T\right)}^{3/2}}{N_{dis}q^{3}f^{2}\lambda_{d}\sqrt{m^{*}}}$$

In (7), $\mu_{dsl}$ is the effect of dislocation scattering on the carrier mobility, $d_{l}$ is the distance between acceptor centers along the dislocation line, *f* is the occupancy rate of the acceptor centers, $N_{dis}$ is the density of dislocations, and $\lambda_{d}={\left[\varepsilon k_{B}T/\left(q^{2}n_{s}\right)\right]}^{1/2}$ is the Debye screening length.

By inserting (5)–(7) into (4), the new 2DEG charge density can be obtained by the following expressions

(8) $$n_{s}={\left(2\beta_{c}V_{th}N_{2-D}\right)}^{2}{\left\{\sum_{i=0,1}\ln\left[1+e^{\left(E_{f}-E_{i}\right)/V_{th}}\right]\right\}}^{2}$$

(9) $$\beta_{c}=\frac{30\sqrt{2\pi }\varepsilon^{2}a^{2}k_{B}T}{N_{dis}q^{2}f^{2}\sqrt{\varepsilon }\sqrt{m^{*}}v_{T}\kappa L_{g}}$$

### 2.3. Calculation of Surface Potential

In order to calculate the Fermi level $E_{f}$, the Schroedinger’s and Poisson’s equations are solved using the depletion approximation as shown by (10)–(11) [12].

(10) $$n_{s}=\frac{\varepsilon }{qd_{d}}\left(V_{g}-V_{off}-E_{f}\right)=\frac{C_{g}}{q}\left(V_{g0}-E_{f}\right)$$

(11) $$E_{0,1}=\gamma_{0,1}n_{s}^{\frac{2}{3}}$$

**Figure 3:**
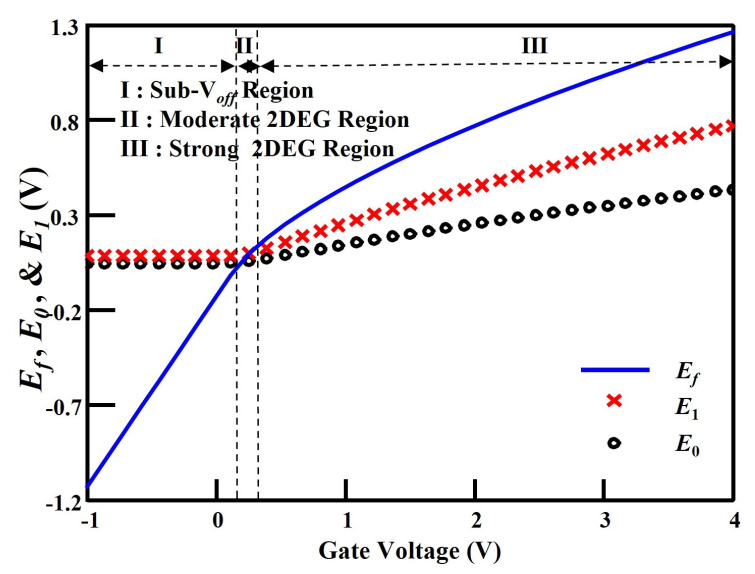
Numerical calculation of $E_{f}$, $E_{0}$ and $E_{1}$ versus gate voltage $V_{g}$. $V_{g}$ varies from −1 to 4 V in the step of 0.1263 V.

In (10), $C_{g}=\varepsilon /d_{d}$ (ε is the permittivity corresponding to InGaAs and $d_{d}$ is the barrier layer thickness) and $V_{g0}=V_{g}-V_{off}$ ($V_{g}$ is the applied gate voltages and $V_{off}$ is the cutoff voltage). In (11), $\gamma_{0}$ and $\gamma_{1}$ are constants, obtained from experiments [13]. The system of (8)–(11) can be iteratively solved through numerical calculation methods. However, these approaches are not suitable for circuit simulation as they are too costly in terms of computation time [3]. Thus, an approximate analytical solution is proposed. The variations of $E_{f}$, $E_{0}$ and $E_{1}$ versus gate bias are depicted in Figure 3, which are numerically solved the above-mentioned equation system. As shown in Figure 3, the curve is segmented into three different regions. The segmentation region is determined by the position of the Fermi level $E_{f}$ relative to the energy levels $E_{0}$ and $E_{1}$. Among them, region I is called the Sub-$V_{off}$ region, in which the gate bias voltage is less than the cutoff voltage (i.e., $V_{g}$ < $V_{off}$). In this region, where $\left|E_{f}\right|\gg E_{0,1}$ and $n_{s}$ is small, the analytical expression for $E_{f}$ (called $E_{f,sub-voff}$) is

(12) $$E_{f,sub-voff}=V_{g0}-4q/C_{g}{\left(2\beta_{c}V_{th}N_{2-D}\right)}^{2}e^{2V_{g0}/V_{th}}$$

Regions II and III are called moderate and strong 2DEG regions, respectively, where the gate bias voltage must be larger than the cutoff voltage (i.e., $V_{g}$ > $V_{off}$).

In the moderate 2DEG region ($E_{f}$ < $E_{0}$, $E_{f}$ > $E_{1}$), where heightened electron occupancy causes an increase in $n_{s}$, the analytical expression for $E_{f}$ (referred to as $E_{f,II}$) is

(13) $$E_{f,II}=V_{g0}\left(\frac{\frac{V_{th}}{2}\ln\left(\beta V_{g0}\right)+\gamma_{0}{\left(\frac{C_{g}V_{g0}}{q}\right)}^{\frac{2}{3}}}{V_{g0}+\frac{V_{th}}{2}+\frac{2}{3}\gamma_{0}{\left(\frac{C_{g}V_{g0}}{q}\right)}^{\frac{2}{3}}}\right)$$

where $\beta =\frac{C_{g}}{{\left(2\beta_{c}V_{th}N_{2-D}\right)}^{2}q}$.

In the strong 2DEG region, where $E_{f}$ > $E_{0,1}$ and $n_{s}$ saturates, the analytical expression for $E_{f}$ (called $E_{f,III}$) is

(14) $$E_{f,III}=V_{g0}\frac{\beta k_{f}V_{th}{\left(V_{g0}\right)}^{\frac{1}{2}}+\gamma_{0}{\left(\frac{C_{g}V_{g0}}{q}\right)}^{\frac{2}{3}}}{V_{g0}+\beta \frac{k_{f}}{2}V_{th}{\left(V_{g0}\right)}^{\frac{1}{2}}+\frac{2}{3}\gamma_{0}{\left(\frac{C_{g}V_{g0}}{q}\right)}^{\frac{2}{3}}}$$

where $k_{f}=\beta_{c}N_{2-D}V_{th}/{\left(C_{g}/q\right)}^{1/2}$.

For region II and region III, there is only a single expression for $E_{f}$ (called $E_{f,above}$), which is

(15) $$E_{f,above}=V_{g0}\left(1-H\left(V_{g0}\right)\right)$$

(16) $$H\left(V_{g0}\right)=\frac{V_{g0}+V_{th}\left[1-\frac{1}{2}\ln\left(\beta V_{gon}\right)-\frac{V_{go}}{2V_{got}}\right]-\frac{\gamma_{0}}{3}{\left(\frac{C_{g}V_{g0}}{q}\right)}^{\frac{2}{3}}}{V_{g0}\left(1+\frac{V_{th}}{2V_{got}}\right)+\frac{2\gamma_{0}}{3}{\left(\frac{C_{g}V_{go}}{q}\right)}^{\frac{2}{3}}}$$

$V_{gon}$ and $V_{got}$ are given by the interpolation expression as

(17) $$V_{gox}=V_{go}\alpha_{x}/\sqrt{V_{go}^{2}+\alpha_{x}^{2}}$$

where *x* is taken as *n* or *t*, $\alpha_{n}=e^{2}/\beta$ and $\alpha_{t}=V_{g0}^{1/2}/\beta k_{f}$.

For the compact model, a unified expression for $E_{f}$ that is able to cover the entire range (i.e., all three regions) of the gate bias voltage is required. To meet this requirement, we have combined the expressions of (12), (15), and (16) to form a unified continuous expression for $E_{f}$ (called $E_{f,unified}$) as

(18) $$E_{f,\;\mathrm{unified}\;}=V_{g0}-\frac{V_{th}\ln\left(1+e^{V_{g0}/V_{th}}\right)}{1/H\left(V_{g0,eff}\right)+C_{g}/\left(qN_{p2d}\right)e^{-V_{g0}/V_{th}}}$$

Here, H($V_{g0,eff}$) tends to infinity when $V_{g}$ < $V_{off}$ while it is equal to H($V_{g0}$) when $V_{g}$ > $V_{off}$ and $N_{p2d}={\left(4\beta_{c}N_{2-D}\right)}^{2}V_{th}$.

However, according to our experiments, in the region where $V_{g}$ is close to $V_{off}$, the error of $E_{f,unified}$ is in the unit of millivolts. In order to improve its accuracy in this area, the Householder’s method [14] is employed. After applying this method, the final refined solution of $E_{f}$ is

(19) $$E_{f}=E_{f,\;\mathrm{unified}\;}-\frac{x}{z}\left(1+\frac{xr}{2z^{2}}\right)$$

The quantities *x* and *z* are defined in Table 1 with $k_{0,1}$, $V_{gef}$, $\xi_{0,1}$, $r_{0}$, $r_{1}$, and *r*. The refinement of (19) is conducted two times to ensure good accuracy for $E_{f}$. Knowing the Fermi potential, the SP φ can be calculated by $\varphi =E_{f}+V_{x}$, where $V_{x}$ is the channel voltage that equals to $V_{s}$ at source and $V_{d}$ at drain. Based on the definition, the potentials at the source ($\varphi_{s}$) and drain ($\varphi_{d}$) are determined.

### 2.4. Drain Current Model

The surface potential calculated at the source and drain ends is used to calculate the drain flux and current. The flux through the channel to the drain can be calculated as

(20) $$a_{D}=-\mu_{eff}n_{s}E_{x}-D\frac{dn_{s}}{dx}=\mu_{eff}n_{s}\frac{d\varphi }{dx}-D\frac{dn_{s}}{dx}$$

$\mu_{eff,sat}$ is the effective mobility including mobility degradation and velocity saturation caused by other scattering mechanisms except dislocation scattering. $E_{x}$ stands for the electric field along the channel and $E_{x}=-d\varphi /dx$. *D* is conventional diffusion coefficient with $D=\mu_{eff,sat}V_{th}$ obtained by the Einstein relation. Substituting (10) into (20) to get

(21) $$a_{D}=\mu_{eff,sat}\frac{C_{g}}{q}\left(V_{g0}-\varphi +V_{th}\right)\frac{d\varphi }{dx}$$

By integrating (21), we can obtain

(22) $$a_{D}=\frac{\mu_{eff,sat}}{L_{g}}\frac{C_{g}}{q}\left(V_{g0}+V_{th}-\varphi_{m}\right)\varphi_{ds}$$

where $\varphi_{ds}=\varphi_{d}-\varphi_{s}$ and $\varphi_{m}=\left(\varphi_{s}+\varphi_{d}\right)/2$. Based on the flux of the drain, $a_{D}$, the core drain current taking care of the channel length modulation can be written as

(23) $$I_{ds}=qWa_{D}\left(1+lambda\cdot V_{ds}\right)$$

*W* is the gate width. $V_{ds}$ is the drain to source voltage and lambda is the coefficient for the channel length modulation. The vertical field mobility degradation due to ionized impurity scattering and lattice scattering can be modeled as

(24) $$\mu_{eff}=\frac{\mu_{0}}{1+\mu_{a}E_{y,eff}+\mu_{b}E_{y,eff}^{2}}$$

where $\mu_{0}$ is the low field mobility. $\mu_{a}$ and $\mu_{b}$ are the first-order and second-order vertical-field mobility degradation coefficients empirically extracted from experimental data. $E_{y,eff}$ is the effective vertical electric field calculated by Gauss’s law as $E_{y,eff}=qn_{s}/\varepsilon$. With increase in the lateral electric field, part of the carrier energy is scattered by optical phonons, leading to the saturation of the carrier velocity. To illustrate this effect, the effective carrier mobility is modified as

(25) $$\mu_{eff,sat}=\frac{\mu_{eff}}{\sqrt{1+{\left(\mu_{eff}/VSAT\cdot E_{x}\right)}^{2}}}=\frac{\mu_{eff}}{\sqrt{1+THESAT^{2}\varphi_{ds}^{2}}}$$

where $E_{x}=\varphi_{ds}/L$. VSAT is the velocity saturation parameters. THESAT is the coefficient for the velocity saturation which theoretically equals $\mu_{eff}/VSAT\cdot L$.

In short-channel InGaAs HEMT devices, DIBL effect must be taken into account in a compact model. DIBL effect causes an increase in the drain current and a decrease in the threshold voltages. We use the following expression to account for this effect

(26) $$V_{off,DIBL}=V_{off}-\delta V_{ds}$$

Here, δ is the DIBL factor. The total current needs to take into account the self-heating effect and access to region resistances. When the device operates in the high voltage and high current region, the self-heating effect plays an important role in the drain current. The self-heating effect is included with a thermal sub-circuit with thermal resistance and capacitance [15]. The access region resistance can be modeled as in [2].

### 2.5. Capacitance Model

Using the charge density at any point in the channel as (14), the gate charge equation is as follows

(27) $$Q_{g}=-\int_{0}^{L_{g}}WC_{g}\left(V_{g0}-\varphi \left(x\right)\right)dx$$

The drain and source charge is defined by Ward–Dutton partition method, we obtain

(28) $$Q_{d}=\int_{0}^{L_{g}}\frac{x}{L_{g}}Q_{ch}\left(V_{g},V_{x}\right)dx$$

(29) $$Q_{s}=\int_{0}^{L_{g}}\left(1-\frac{x}{L_{g}}\right)Q_{ch}\left(V_{g},V_{x}\right)dx$$

where $Q_{ch}$ is the channel charge. Integrating (27) and (28) from source to drain, the gate and drain charges are the same as those of the ASM-HEMT model [2].

(30) $$\begin{array}{c} Q_{g}=\frac{WC_{g}L_{g}}{\left(V_{g0}-\varphi_{m}+V_{th}\right)}\left[V_{g0}^{2}-V_{g0}\left(\varphi_{d}+\varphi_{s}-V_{th}\right)\right.\\ \left.+\frac{1}{3}\left(\varphi_{d}^{2}+\varphi_{s}^{2}+\varphi_{d}\varphi_{s}\right)-V_{th}\varphi_{m}\right] \end{array}$$

(31) $$\begin{array}{c} Q_{d}=-\frac{WC_{g}L_{g}}{120{\left(V_{g0}-\varphi_{m}+V_{th}\right)}^{2}}\left[12\varphi_{d}^{3}+8\varphi_{s}^{3}+\varphi_{s}^{2}\left(16\varphi_{d}\right.\right.\\ \left.\;\;-5\left(V_{th}+8V_{g0}\right)\right)+2\varphi_{s}\left(12\varphi_{d}^{2}-5\varphi_{d}\left(5V_{th}+8V_{g0}\right)\right.\\ \;\;\left.+10\left(V_{th}+V_{g0}\right)\left(V_{th}+4V_{g0}\right)\right)+15\varphi_{d}^{2}\left(3V_{th}+4V_{g0}\right)\\ \;\;\left.-60V_{g0}{\left(V_{th}+V_{g0}\right)}^{2}+20\varphi_{d}\left(V_{th}+V_{g0}\right)\left(2V_{th}+5V_{g0}\right)\right] \end{array}$$

According to the charge conservation, the source charge is obtained using $Q_{s}=-Q_{g}-Q_{d}$. The intrinsic capacitance can be obtained by using the definition $C_{ij}=-\frac{\partial Q_{s}}{\partial V_{j}}\left(i\neq j\right)$ and $C_{ij}=\frac{\partial Q_{s}}{\partial V_{j}}\left(i=j\right)$, with *i* and *j* corresponding to the device terminals.

## 3. Model Verification and Discussion

Here, the numerical calculations of $E_{f}$ are first performed to verify the analytical solution of the surface potential of the new model. To further validate the entire model, the drain current, capacitance, small- and large-signal model are verified by experimental data.

### 3.1. Comparison to Numerical Simulations

**Figure 4:**
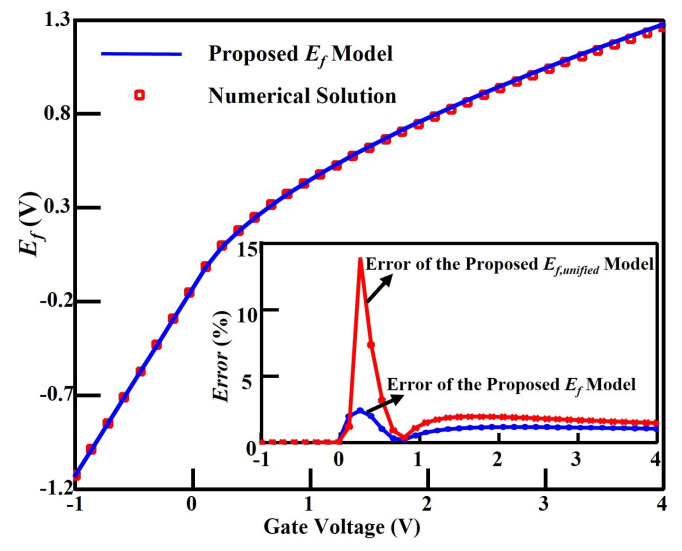
Comparison of the proposed $E_{f}$ model and the numerical solution for the 30-nm InGaAs HEMT devices. The insert shows the deviation of the proposed $E_{f,unified}$ and $E_{f}$ models compared with the numerical solution.

The proposed $E_{f}$ model is simulated for InGaAs HEMT device [16,17]. The comparison of the analytical solution of $E_{f}$ with the numerical solution of (8)–(11) is shown in Figure 4. As one can see, the proposed model agrees well with the numerical calculation by using the parameter values depicted in Table 2. The insert in Figure 4 illustrates the relative error of $E_{f,unified}$ and $E_{f}$ compared with the numerical solution, in which the error is less than 2% over the entire range of the gate bias except for the area around the cutoff voltage.

Since the assumption $E_{f,sub-voff}$≈$V_{g0}$ becomes invalid when $V_{g}$ is very close to $V_{off}$, the error of $E_{f}$ is relatively large in this area, which reaches the maximum value of 13.91%, as proved by the red curve. However, after applying the refinement operation, the maximum error drops to only 2.41%, as shown by the blue curve in the insert. These results demonstrate the necessity of the refinement operation and the accuracy of the proposed $E_{f}$ model.

### 3.2. Comparison to Experimental Data

In order to verify the accuracy of the proposed large-signal model, the device is used with a gate length of 100 nm and a total gate width of 150 μm. The basic performance DC, S parameters, and power characteristics of InGaAs HEMT device were tested.

**Figure 5:**
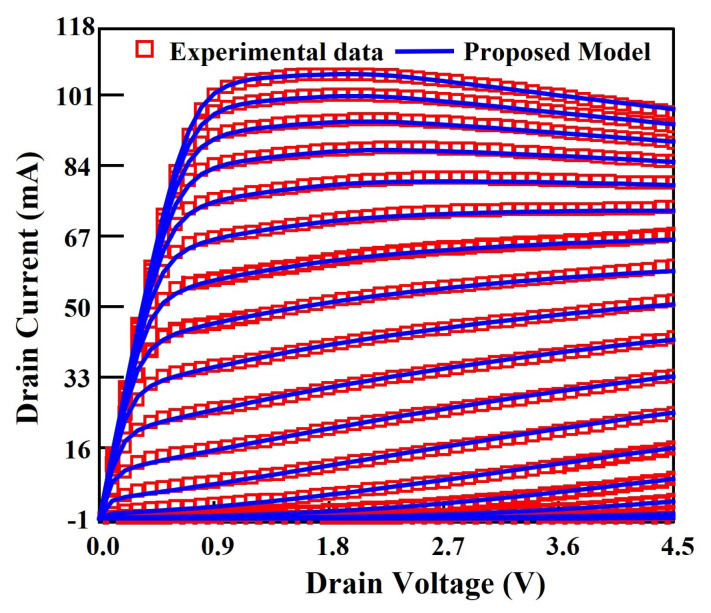
Comparison of the modeled output characteristics with experimental data of the 100 nm InGaAs HEMT device. $V_{gs}$ varies from 2.0 to 0.5 V (bottom to top) in the steps of 0.1 V.

**Figure 6:**
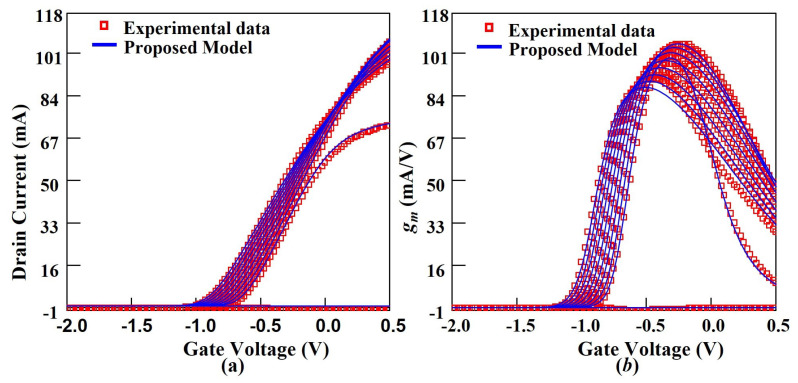
Comparison of the modeled transfer characteristics (**a**) and transconductance (**b**) with experimental data of the 100 nm InGaAs HEMT device. $V_{ds}$ varies from 0 to 4.5 V (bottom to top) in the steps of 0.5 V.

The drain current and capacitance model are implemented in Verilog-A (VA). Then the VA file containing the core model is imported into ICCAP software from Agilent. In order to accurately characterize the S parameter in this experiment, a subcircuit containing parasitic components is used to characterize the parasitic capacitance, inductance, and resistance in the device. The simulation is achieved by calling the Keysight ADS simulator in ICCAP and fitting the model simulations to the test data by adjusting the model parameters. The extracted relevant model parameters of the device are given in Table 3. A comparison of the output characteristics ($I_{ds}$–$V_{ds}$), transfer characteristics ($I_{ds}$–$V_{gs}$) and transconductance ($g_{m}$–$V_{gs}$) are shown in Figure 5, Figure 6a, and Figure 6b, respectively. In these cases, the model and the experimental data are in excellent agreement, indicating a good accuracy of the drain model.

**Figure 7:**
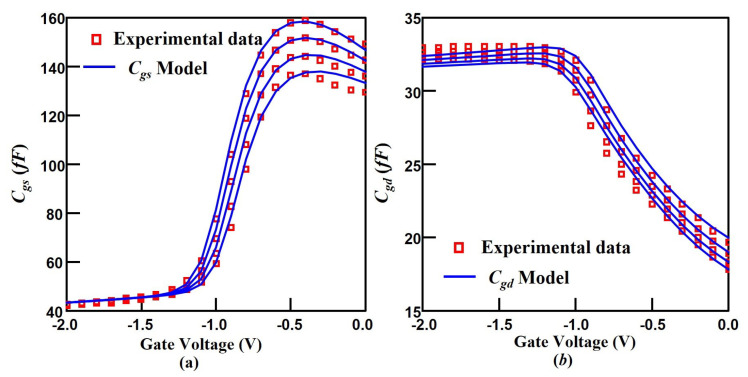
Comparison of the modeled transfer characteristics (**a**) and transconductance (**b**) with experimental data of the 100 nm InGaAs HEMT device. $V_{ds}$ varies from 0 to 4.5 V (bottom to top) in the steps of 0.5 V.

The plots of $C_{gs}$ versus $V_{gs}$ and $C_{gd}$ versus $V_{gs}$ are shown in Figure 7a and Figure 7b, respectively. It is clear from the two plots that the variation of $C_{gs}$ and $C_{gd}$ with the $V_{ds}$ is relatively small, which is due to the fact that the 2DEG charge density is mainly modulated by the $V_{gs}$. At $V_{gs}$ below −1.2 V, the $C_{gs}$ ($C_{gd}$) versus $V_{gs}$ corresponds to the deep depletion region of electrons in the buffer layer, where the electrons in the buffer layer are further depleted after the channel electrons are depleted. The region is mainly characterized by the fringing capacitance. The extracted values of the fringing capacitance for $C_{gs}$ and $C_{gd}$ are 37.26fF and 24.77fF, respectively. Within −1.2 V < −0.4 V, the $C_{gs}$ ($C_{gd}$) versus $V_{gs}$ belongs to the 2DEG depletion region, reflecting the gradual depletion of electrons from the interface to the buffer layer. The steeper the curve in this region, the better the heterojunction performance. At $V_{gs}$ above −0.4 V, it can be observed that $C_{gs}$ tends to decrease. The observed phenomenon is controlled by the gate-source and gate-drain diode. The reason is that the depletion charge is reduced due to the gate-source and gate-drain diodes are turned on. The proposed capacitance model cannot fully cover all trends of capacitance variation with gate and drain voltages. The fitting results at present have basically met the requirements, so more detailed optimization was not performed.

**Figure 8:**
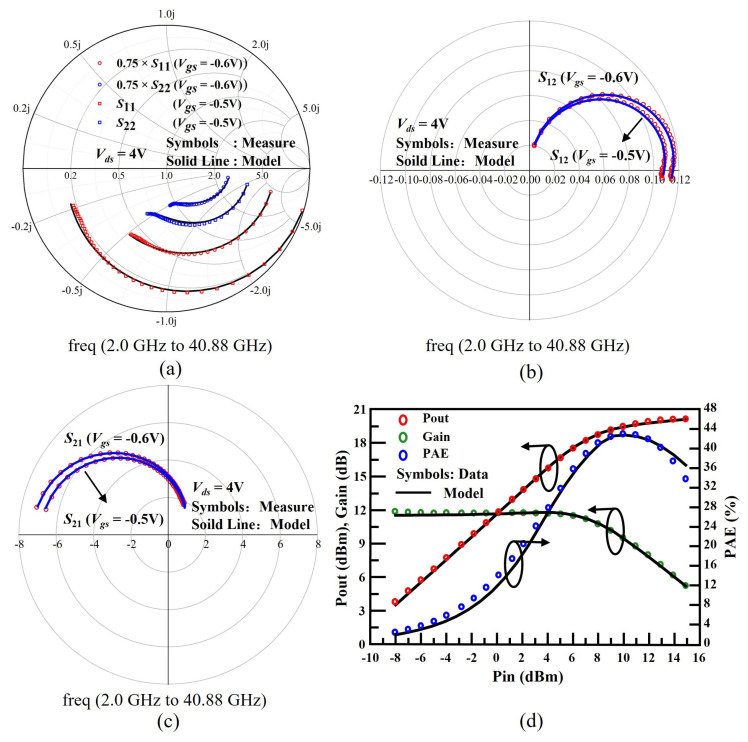
Accurate modeling of small-signal S-parameters for frequency range 2.0 GHz to 40.88 GHz at $V_{ds}$ = 4V and two difference $V_{gs}$ conditions $V_{gs}$ = −0.5 V and $V_{gs}$ = −0.6 V: (**a**) $S_{11}$ and $S_{22}$; (**b**) $S_{12}$; (**c**) $S_{21}$; (**d**) Modeling of large-signal RF output power (Pout), RF power gain and PAE (%) as the input power Pin is varied when input signal frequency is 29 GHz. Pin varies from −8 to 14.9 dBm in the step of 0.95417 dBm.

In Figure 8a–c, S-parameters measured from 2.0 GHz to 40.88 GHz at two gate bias points. In Figure 8d, large signal RF results varied with RF input power are shown. The simulated results have been performed using ADS software from Agilent. The variation in output power Pout, Power Gain, and Power-added efficiency (PAE) for $V_{ds}$ = 4 V and $I_{ds}$ = 23 mA condition in Figure 8d. It is observed that simulated results of these key figures closely match with the experimental data with the help of the core and various physical effects model.

## 4. Conclusions

A new analytical model for quasi-ballistic transport in InGaAs HEMTs is proposed. A new 2DEG charge density is derived using the one-flux method. The transmission coefficient, accommodating the dislocation scattering, is introduced. The new derived charge density is employed to conduct the analytical calculation for the Fermi level $E_{f}$ and SP. A drain current model including important real device phenomena is developed. In addition, the gate-source and gate-drain capacitances are both obtained analytically. The proposed $E_{f}$ model agrees well with the numerical solution, which demonstrates its high efficacy. The drain, capacitance, and small- and large-signal model are in excellent agreement with the experimental data of the 100 nm InGaAs HEMT device.

## Figures and Tables

**Table 1:** Expression for quantities used in (19).

Quantity	Expression
$k_{0,1}$	$\gamma_{0,1}{\left(C_{g}/q\right)}^{\frac{2}{3}}$
$V_{gef}$	$V_{g}-V_{off}-E_{f}$
$\xi_{0,1}$	$\exp\left[\left(E_{f,\;\mathrm{unified}\;}-k_{0,1}V_{\mathrm{gef}\;}^{\frac{2}{3}}\right)/V_{th}\right]$
*x*	$C_{g}V_{gef}/q-{\left[\sum_{i=0}^{1}2k_{f}V_{th}N_{2-D}\ln\left(\xi_{i}+1\right)\right]}^{2}$
*z*	$-\frac{C_{g}}{q}-\sum_{i=0}^{1}8k_{f}^{2}V_{th}N_{2-D}^{2}\frac{\ln\left(\xi_{i}+1\right)}{1+\xi_{i}^{-1}}\left(1+\frac{2}{3}k_{i}V_{gef}^{-\frac{1}{3}}\right)$
$r_{0}$	$\left[{\left(1+\frac{2}{3}k_{i}V_{gef}^{-\frac{1}{3}}\right)}^{2}\left(1+\ln\left(\xi_{i}+1\right)/\xi_{i}\right)\right]/V_{th}$
$r_{1}$	$\left(1+\xi_{i}^{-1}\right)\ln\left(\xi_{i}+1\right)k_{i}V_{gef}^{-\frac{4}{3}}$
*r*	$8k_{f}^{2}V_{th}N_{2-D}^{2}\sum_{i=0}^{1}\left(r_{0}+r_{1}\right)/{\left(1+\xi_{i}^{-1}\right)}^{2}$

**Table 2:** Parameter values used in the model and numerical calculation for 30 nm InGaAs HEMT devices.

Parameter	Values	Parameter	Values
$L_{g}$ [nm]	30	*d* [nm]	24
$V_{off}$ [mV]	126.3 [18]	$v_{T}$	$2.65\times 10^{7}$
$N_{dis}$ [cm^−2^]	$10^{10}$ [19]	$d_{l}$ [nm]	4.5 [19]
*f*	0.5	$m^{*}$	0.035 $m_{0}$
ε	12.65 $\varepsilon_{0}$	$\gamma_{0,1}$	$2.26\times 10^{7};4.0\times 10^{7}$ [13]

**Table 3:** The extracted model parameter values for the 100 nm InGaAs HEMT device.

Parameter	Values	Parameter	Values
$V_{off}$ [V]	$-573.2\times 10^{-3}$	VSAT [m/s]	$252.4\times 10^{3}$
$\mu_{0}$ [m^2^/(V·s)]	$959.8\times 10^{-3}$	$v_{T}$ [cm/s]	$2.476\times 10^{7}$
$\mu_{a}$ [V^−1^]	$47.69\times 10^{-9}$	$N_{dis}$ [cm^−2^]	$13.05\times 10^{10}$
$\mu_{b}$ [V^−2^]	$357.3\times 10^{-18}$	$d_{l}$ [nm]	4.875
lambda [V^−1^]	$3.971\times 10^{-6}$	*f*	0.1642
δ	$78.94\times 10^{-3}$	κ	1.755
THESAT [V^−2^]	13.88		

## Data Availability

A commercial common process.
